# Manual of Travel Medicine and Health

**DOI:** 10.3201/eid1007.031004

**Published:** 2004-07

**Authors:** Drew L. Posey

**Affiliations:** * Centers for Disease Control and Prevention, Atlanta, GA, USA

**Keywords:** book review, travel medicine

## Abstract

Manual of Travel Medicine and Health

Although the field of travel medicine is relatively young, the number of textbooks about the field is growing. This international trio of authors from Switzerland, the United States, and Singapore recently published the second edition of their textbook, which adds new chapters and updates epidemiologic information. Because of the complexity of travel medicine, good resources for clinicians and travelers are needed. This edition represents a welcome addition to the library of travel medicine.

The main audience for this textbook is travel medicine physicians. Like the first edition, it is designed to be a reference book. Although small enough to fit in a pocket of a white coat, this paperback is very readable and comes with an easy-to-use CD.

Part 1 of the book, Basics, provides an overview of general topics for physicians to discuss with their traveling patients. The authors encourage a comprehensive strategy, one that discusses prevention measures such as vaccines and their appropriate uses. Appendix C is an excellent table that lists the required and recommended vaccinations for each country. The text also provides excellent information for travelers in varied situations, such as pilgrims to the Hajj, migrants, pregnant women, international adoptees, athletes, and persons who are immunocompromised.

In addition to providing current information on immunizations, the authors provide thorough information on malaria, including some individual country maps displaying areas of risk. Although the malaria review is comprehensive, caution should be exercised when deciding not to provide prophylaxis for travelers to a country where malaria is endemic. Part 2, Infectious Health Risks and Their Prevention, is the familiar chronicle of travel-related infectious diseases. This section includes numerous maps and tables describing the epidemiology of the diseases. The authors have updated this part by adding several diseases, including severe acute respiratory syndrome.

The book provides pertinent information on travelers' medical kits, water disinfection, and noninfectious health risks such as high altitude, arctic travel, diving, jet lag, and ultraviolet radiation. New for this edition are informative chapters on deep vein thrombosis and pulmonary embolism and in-flight accidents.

Another strength of this work is the section on posttravel medical treatment. This chapter presents concise guidelines for the clinician who is treating posttravel patients (with diarrhea, fever, malaria, dermatologic disorders, eosinophilia, sexually transmitted diseases) or screening expatriates after prolonged stays in tropical regions. A particularly useful feature is the dosing recommendations, many of which are for infrequently used drugs.

In conclusion, the Manual of Travel Medicine and Health, Second Edition, should be a useful textbook for travel medicine physicians and those in training who want to learn more about the field. While the traditional topics are covered in customary detail, the strength of the book is its comprehensiveness and portability, providing a convenient reference.

